# An immediate effect of PNF specific mobilization on the angle of trunk rotation and the Trunk-Pelvis-Hip Angle range of motion in adolescent girls with double idiopathic scoliosis—a pilot study

**DOI:** 10.1186/s13013-017-0132-0

**Published:** 2017-09-06

**Authors:** A. Stępień, K. Fabian, K. Graff, M. Podgurniak, A. Wit

**Affiliations:** 1 Department of Rehabilitation, Józef Piłsudski University of Physical Education, Warsaw, Poland; 2 Regional Children’s Hospital, Jastrzębie Zdrój, Poland; 3 Department of Physiological Sciences, University of Life Science, Warsaw, Poland

**Keywords:** Idiopathic scoliosis, PNF, Angle of trunk rotation, Rotation, Physiotherapy

## Abstract

**Background:**

Impairment of spine rotation is a key concept in several theories explaining the pathogenesis and progression of scoliosis. In previous studies, a more limited range of motion in scoliotic girls compared to their non-scoliotic peers was noted. The Trunk-Pelvis-Hip Angle measurement is a test used to assess the range of motion in the trunk-pelvis-hip complex in the transverse plane. The aim of this study was to assess an immediate effect of Proprioceptive Neuromuscular Facilitation specific mobilization (mPNF) on the angle of trunk rotation and Trunk-Pelvis-Hip Angle range of motion in adolescent girls with double scoliosis.

**Methods:**

The study was conducted on 83 girls aged 10 to 17 years (mean 13.7 ± 1.9) with double idiopathic scoliosis consisting of a right-sided thoracic curve (mean 25.1° ± 13.9°) and a left-sided thoracolumbar or lumbar curve (mean 20.8° ± 11.4°). The angle of trunk rotation and Trunk-Pelvis-Hip Angle were measured at baseline and after PNF mobilization. Bilateral lower limb patterns of Proprioceptive Neuromuscular Facilitation were used in combination with the “contract–relax” technique and stimulation of asymmetrical breathing. In the statistical analysis, the SAS rel. 13.2 software was used. Preliminary statistical analysis was performed using descriptive statistics. According to Shapiro-Wilk criterion of normality, the Wilcoxon test was used to compare paired samples. Next, the data was analyzed using multivariate GLM models.

**Results:**

In adolescent girls with double scoliosis, significant differences between the left and right side of the body concerning the Trunk-Pelvis-Hip Angle ranges were noted. A single, unilateral PNF mobilization significantly decreased the angle of trunk rotation in the thoracic (*p* < 0.001) and lumbar spine (*p* < 0.001). Unilateral PNF mobilization also increased the Trunk-Pelvis-Hip Angle ranges on the left (*p* < 0.001) and right (*p* < 0.001) side significantly.

**Conclusions:**

Unilateral PNF mobilization led to a decrease in the angle of trunk rotation, improvement in the range of motion, and the symmetry of mobility in the transverse plane in the trunk-pelvis-hip complex in adolescent girls with double idiopathic scoliosis. The effects should be treated only as immediate. Further studies are required to determine long-term effects of PNF mobilization on the spinal alignment.

**Trial registration:**

ISRCTN11750900.

## Background

Idiopathic scoliosis is one of the most common spinal deformities in children and adolescents. It is defined as three-dimensional torsional deformity characterized by lateral deviation of the spine equal to or greater than 10°, vertebral rotation and reduced normal thoracic kyphosis [1, 2]. According to the literature, adolescent idiopathic scoliosis (AIS) affects 0.93 to 12% of adolescents aged 10–18 years and is more common in girls [2, 3].

Vertebral rotation with the associated rib hump and/or lumbar hump is a major sign of scoliosis. That is why measurement of the angle of trunk rotation (ATR) with a scoliometer is an important element of scoliosis examination. To date, however, a standard physical examination has not included an assessment of the spine rotation range. It may stem from the fact that scoliotic individuals demonstrate generalized joint hypermobility significantly more often [4, 5]. On the other hand, previous studies showed a more limited range of motion (ROM) in the transverse plane in scoliotic girls compared to their non-scoliotic peers [6–8]. It was noted that the limitation in the range of rotation increased with the Cobb angle [6]. The greatest differences in ROM between the left and right side of the body were observed in girls with double scoliosis [7]. The Trunk-Pelvis-Hip Angle (TPHA) measurement is a test used to assess the range of motion in the trunk-pelvis-hip complex in the transverse plane. The TPHA test showed rotational asymmetry in girls with double scoliosis [8]. According to some theories, an impairment of spine rotation is considered a factor predisposing to the development and progression of scoliosis [9, 10].

Proprioceptive Neuromuscular Facilitation (PNF) method is used in patients with various motor problems and offers a large number of movement patterns, procedures, and techniques, including effective techniques to improve ROM [11–13]. Precisely described movement patterns allow the therapist to act selectively on particular parts of the musculoskeletal system, including the spine [12]. To date, the effectiveness of PNF in the conservative treatment of individuals with idiopathic scoliosis has not been confirmed.

The aim of this study was to assess an immediate effect of PNF specific mobilization on the ATR and the TPHA range of motion in adolescent girls with double scoliosis.

## Methods

The study was approved by the Senate Research Ethics Committee at Jozef Pilsudski University of Physical Education in Warsaw, Poland, SKE 01-04/2015.

### Study subjects

Scoliotic girls who visited one of the three centers specializing in the conservative treatment of scoliosis were consecutively enrolled in the study which lasted 6 months. The inclusion criteria were as follows: female, double idiopathic scoliosis consisting of a right-sided thoracic curve and a left-sided lumbar/thoracolumbar curve diagnosed on antero-posterior radiogram, absence of systemic diseases, age 10–17 years, and participation consent. Girls with other types of scoliosis, a spinal curvature with a Cobb angle of less than 10°, with pain or a history of traumatic injury were excluded from the study.

The girls and their parents or legal guardians were informed about the aims of the study and signed the consent to participate in the study and publish anonymous data form.

### Measurement methods

The study was performed by three physiotherapists who were experienced in treating scoliosis and were trained and certified in PNF. The physiotherapists were also trained regarding the application of the TPHA test.

**Fig. 1:**
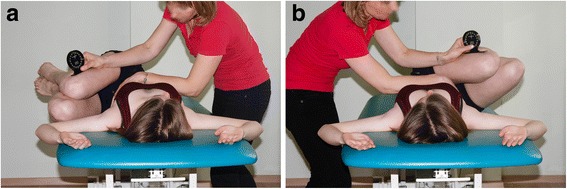
**a** The Trunk-Pelvis-Hip Angle on the *left* (TPHAleft). **b** The Trunk-Pelvis-Hip Angle on the *right* (TPHAright)

At one session, the girls underwent a physiotherapy examination twice, at the beginning and at the end of the session. The examination, carried out by a physiotherapist, included test 1, i.e., a standard measurement of the ATR, and test 2, i.e., an assessment of the active ROM in the trunk-pelvis-hip complex using the TPHA test (Fig. 1a, b). After the first examination, the girls underwent mobilization using the bilateral lower limb PNF patterns (mPNF) and were again assessed (test 1 and test 2) to find out whether the mobilization had any effect on the ATR and TPHA.

The ATR (test 1) was measured with a scoliometer (Orthopedic Systems Inc. OSI 1995), which showed excellent reliability in the previous studies [14–16]. One measurement was performed on the thoracic and lumbar segments of the spine with the subject standing. The range of motion in the TPHA (test 2) was assessed with a Rippstein plurimeter (Rippstein, Switzerland). The assessment of the TPHA test reliability demonstrated excellent agreement of measurements—the inter-rater reliability for three investigators was 0.97 (THPAleft) and 0.98 (TPHAright) and the intraobserver reliability was 0.87 (TPHAleft) and 0.81 (TPHAright) [8].

The TPHA measurements were performed on an adjustable-height table with the subject in a supine position, arms perpendicular to the trunk, and elbows flexed at a 90° angle. Next, lower limbs were flexed at the hips and knees until the sacral bone was involved in the motion and the lumbar segment remained on the surface of the table. Standing at the subject’s side, the examiner stabilized her chest with his or her own forearm kept across the chest at the level of the costal arches and measured the ROM with the other hand. The plurimeter base was held along the long axis of the femur on the lateral aspect of the thigh, at the knee fissure. The subject was asked to move the flexed lower limbs towards the elbow opposite to the examiner. The motion of the lower limbs was stopped when the examiner felt the ribs move under his or her forearm or the subject experienced pain. The actual measurement was performed at the upper ROM limit, 5 s after it had been reached. Before the measurement, the plurimeter was reset to zero in relation to the surface. The angle of the hip position below the surface level was marked as “–”and that above the surface level as “+”. The measurements were performed in triplicate, alternately on the left and right side. The highest ROM values were used in the statistical analysis.

### Mobilization techniques

The therapeutic session consisted of unilateral PNF specific muscle mobilization (mPNF), which was safe and painless. The direction of mPNF was determined on the basis of the results of previous research which revealed limitations of the range of motion in the TPHAright compared to the TPHAleft both in girls with double scoliosis and healthy girls [8]. Bilateral lower limb patterns (flexion to the right and extension to the left) were used in subjects in a supine position with a stable chest in combination with the “contract–relax” technique and stimulation of asymmetrical breathing. The subject had to maintain muscle tension for 5 s three times in succession and then actively increase ROM. This cycle was repeated three times at 10-s intervals. The last phase of mobilization consisted of 10 active movements of lower limbs towards mobilization and asymmetrical breathing—5 slow inspirations and expirations (Table 1). The total duration of mobilization was approximately 3 min.

**Table 1:**
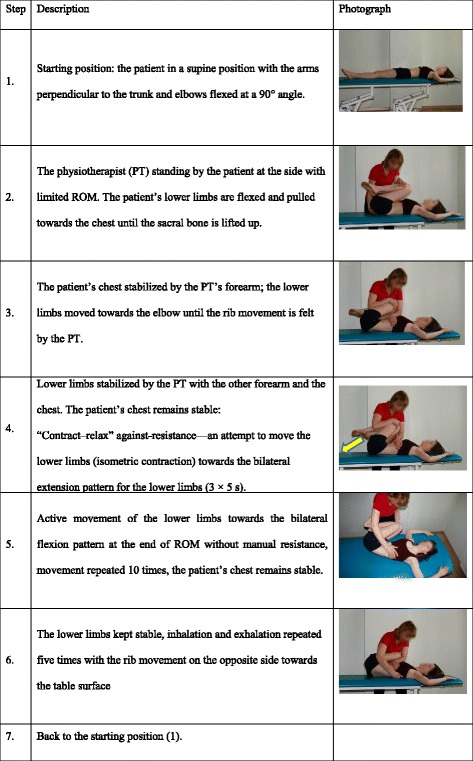
PNF mobilization method

*mPNF* PNF mobilization, *PT* physiotherapist, *ROM* range of motion

After mPNF, the measurement of the ATR and TPHA was repeated and compared with the measurements obtained at baseline.

In the statistical analysis, the SAS rel. 13.2 software was used. Preliminary statistical analysis was performed using descriptive statistics. According to Shapiro-Wilk criterion of normality, the Wilcoxon test was used to compare paired samples. Next, the data was analyzed using multivariate GLM models. The analysis of power and sample size was performed in the SAS System. As shown in the sample size analysis based on a pilot data and confirmed by the results of the main study, the power of the tests used in this manuscript should exceed 0.80.

## Results

In the period of 6 months, 87 girls with double scoliosis were qualified for participation in the study. Four girls did not come to the meeting with a physiotherapist. Ultimately, the study was performed on 83 girls with double idiopathic scoliosis with different levels of deformity diagnosed according to the recommended classification [2]. The basic characteristics of the group are presented in Table 2.

**Table 2:** Characteristics of the study group (age, weight, height, BMI, Th Cobb, L Cobb)

	Mean (*N* = 83)	SD	Min.	Max.
Age (years)	13.7	1.9	10	17
Weight (kg)	51.8	10.9	23	75
Height (m)	1.62	9.5	1.29	1.77
BMI (kg/m^2^)	19.5	2.7	13.8	24.7
Th Cobb (°)	25.1	13.9	10	82
L Cobb (°)	20.8	11.4	10	53

*BMI* body mass index, *N* number, *Th Cobb* Cobb measurements in the thoracic spine, *L Cobb* Cobb measurements in the lumbar spine, *SD* standard deviation, *SE* standard error, *Min* minimum, *Max* maximum

When the classification by a degree of spinal curvature was used in compliance with the guidelines [2], 24 girls (28.9%) were found to have low scoliosis (10°–15°); 16 girls (19.3%), low to moderate scoliosis (16°–24°); 19 girls (22.9%), moderate scoliosis (25°–34°); 16 girls (19.3%), moderate to severe scoliosis (35°–44°); and 8 girls (9.6%) had severe and very severe scoliosis. The Risser grade was as follows: 0 in 8 girls (9.6%), 1 in 13 girls (15.7%), 2 in 10 girls (12.0%), 3 in 16 girls (19.3%), 4 in 27 girls (32.5%), and 5 in 9 girls (10.8%). Twenty-five subjects (30.1%) wore a brace. Most study participants (*n* = 62, 74.7%) underwent some physiotherapy, while 21 girls did not receive any physiotherapeutic treatment. In this group, 11 girls had low scoliosis.

Mean values of the ATR and TPHA obtained before and after mPNF are presented in Table 3. The comparison of the initial values of the TPHA on both sides of the body in the entire study group revealed that the TPHAleft was significantly greater than the TPHAright (*p* < 0.001).

**Table 3:** Values of the ATR in the thoracic/lumbar part of the spine and the TPHA before and after mPNF in girls with double scoliosis

	ATRT ± SEM	ATRL ± SEM	TPHAleft ± SEM	TPHAright ± SEM	TPHAleft vs TPHAright
Before mPNF	7.96° ± 0.75°	4.90° ± 0.39°	− 7.65° ± 0.94	+ 1.82° ± 1.18°	*p* < 0.001
After mPNF	5.71° ± 0.61°	3.23° ± 0.33°	− 12.48° ± 0.47	− 9.78° ± 0.85°	*p* < 0.001
Significance	*p* < 0.001	*p* < 0.001	*p* < 0.001	*p* < 0.001	

Significant difference *p* < 0.05

*ATR* angle of trunk rotation, *ATRT/ATRL* angle of trunk rotation in the thoracic/lumbar part of the spine; *TPHA* Trunk-Pelvis-Hip Angle; *TPHAleft/TPHAright* TPHA to the left/right; *mPNF* PNF mobilization; SEM standard error of measurement

The mPNF significantly reduced the ATR in the thoracic (ATRT, *p* < 0.001) and lumbar spine (ARTL, *p* < 0.001).

**Fig. 2:**
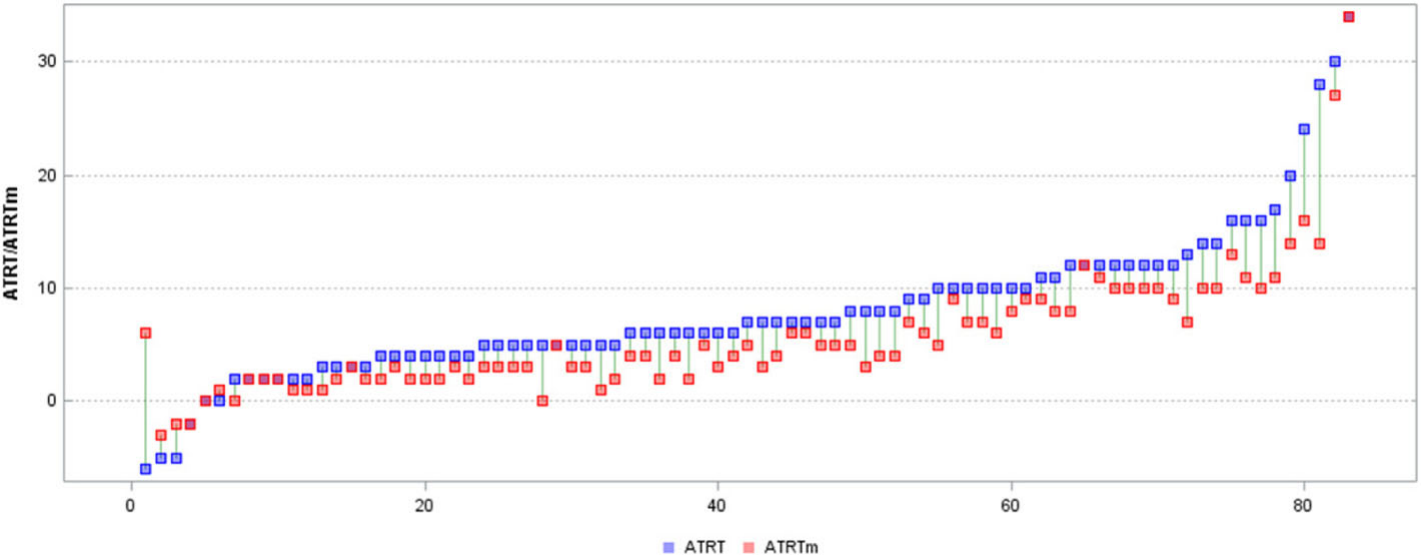
Measurements (°) of the ATRT and ATRTm in individual participants (*ATRT* angle of trunk rotation in the thoracic spine before mPNF, *ATRTm* angle of trunk rotation in the thoracic spine after mPNF, *mPNF* PNF mobilization)

**Fig. 3:**
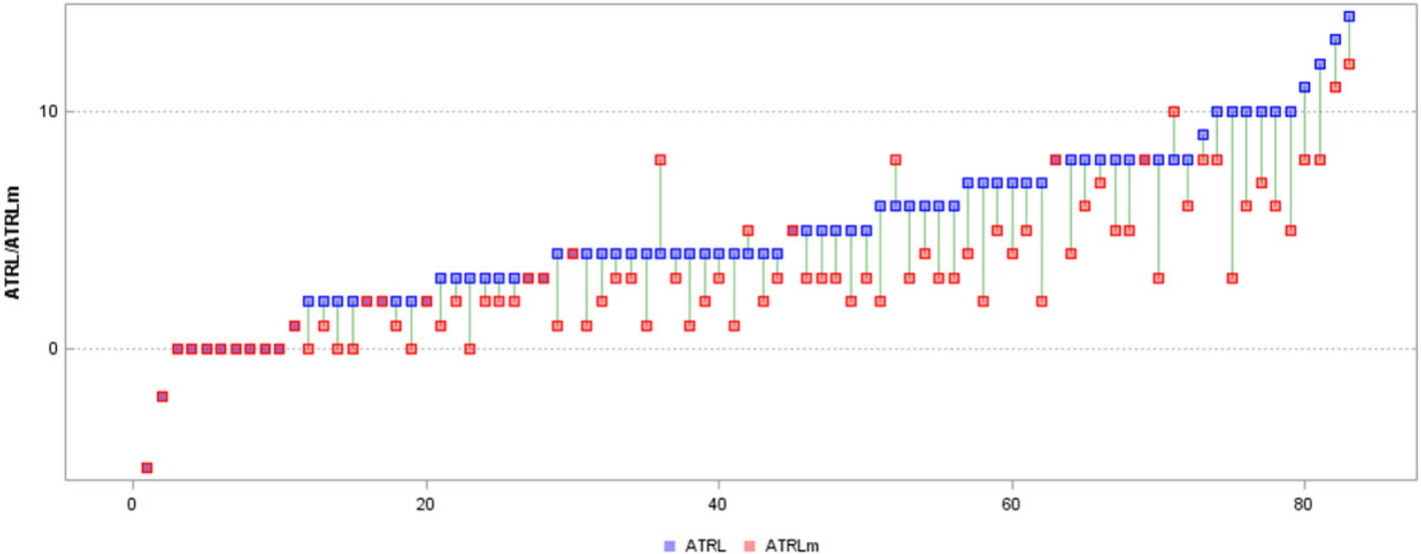
Measurements (°) of the ATRL and ATRLm in individual participants (*ATRT* angle of trunk rotation in the lumbar spine before mPNF, *ATRLm* angle of trunk rotation in the thoracic spine after mPNF, *mPNF* PNF mobilization)

In the majority of the subjects, the decrease in the ATR in the thoracic (ATRT) and lumbar (ATRL) segment was obtained. In 35 girls, ATR values decreased by at least 2° both in the thoracic and in the lumbar segment. A simultaneous analysis of both curve angles revealed that in 55% of the subjects, one of the ATR values changed by at least 3°. An improvement in the ATR in the thoracic or lumbar spine from 0 to 2 was noted in 37 subjects. The biggest changes in the ATR were noted in the thoracic segment of the spine in girls with scoliosis deeper than 35°. Changes in the ATRT (Fig. 2) and ATRL (Fig. 3) occurring after mPNF in particular girls are presented below.

**Fig. 4:**
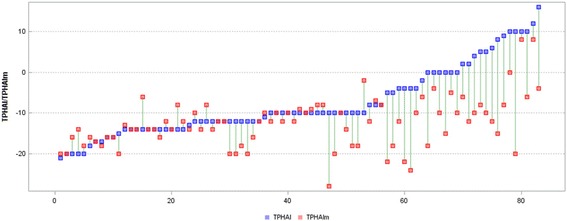
Measurements (°) of the TPHAleft and mTPHAleft in individual participants (*TPHAleft* Trunk-Pelvis-Hip Angle on the left before mPNF, *mTPHAleft* Trunk-Pelvis-Hip Angle on the left after mPNF, *mPNF* PNF mobilization)

**Fig. 5:**
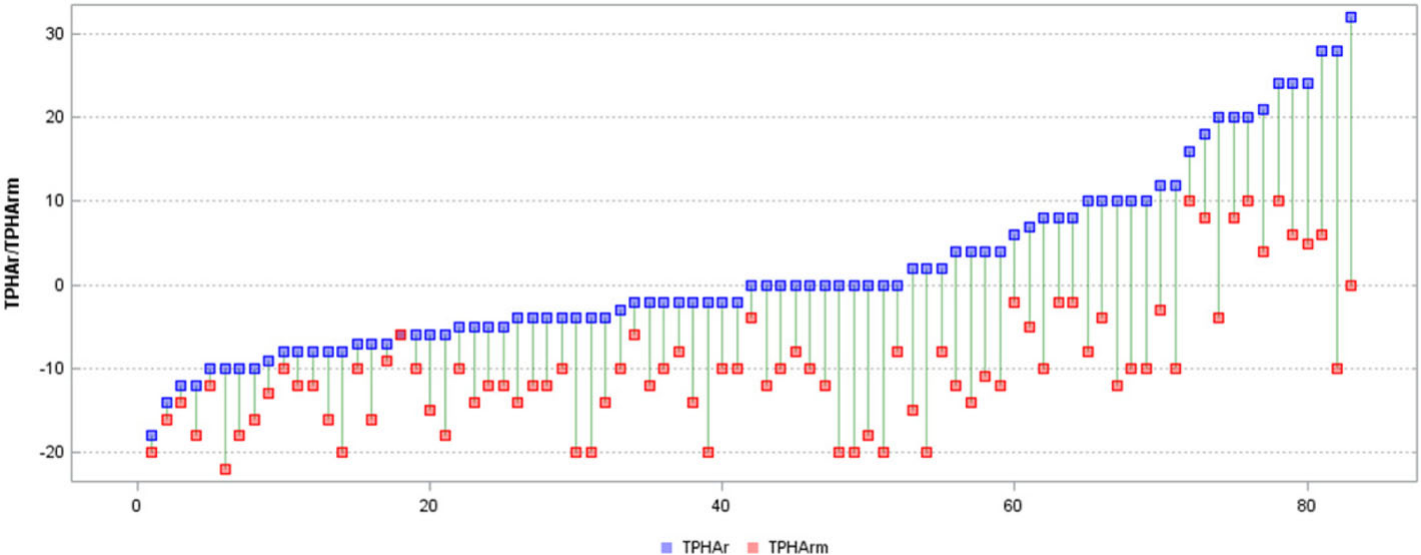
Measurements (°) of the TPHAright and mTPHAright in individual participants (*TPHAright* Trunk-Pelvis-Hip Angle on the right before mPNF, *mTPHAleft* Trunk-Pelvis-Hip Angle on the *right* after mPNF, *mPNF* PNF mobilization)

The mPNF significantly increased the ROM in both the mTPHAleft (TPHAleft measured after mobilization) and mTPHAright (TPHAright measured after mobilization). After mobilization, the difference between the values of the mTPHAleft and mTPHAright decreased although it remained significant (*p* < 0.001). Figures 4 and 5 present the TPHAleft and TPHAright values before and after mobilization in particular girls.

**Fig. 6:**
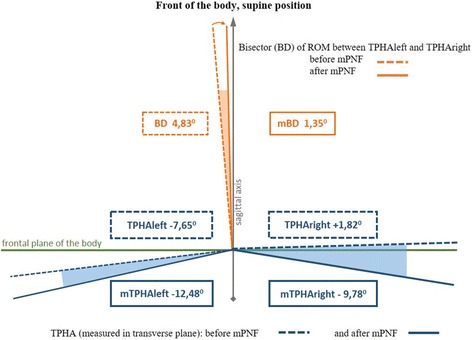
The bisector BD of the angle between the TPHAleft and TPHAright before and after mPNF (*ROM* range of motion, *mPNF* PNF mobilization, *TPHAleft* Trunk-Pelvis-Hip Angle on the left before mPNF, *mTPHAleft* Trunk-Pelvis-Hip Angle on the *left* after mPNF, *TPHAright* Trunk-Pelvis-Hip Angle on the *right *before mPNF, *mTPHAright* Trunk-Pelvis-Hip Angle on the right after mPNF, *BD* bisector deviation angle from the sagittal axis of the body, *mBD* BD after mPNF)

In 12 out of 83 girls, no lower values of the TPHAright compared to the TPHAleft before the mobilization were revealed. Despite the lack of movement limitation, unilateral mobilization decreased the ATR and changed the ranges of motion in all the girls from this subgroup.

In order to reflect the changes in the TPHA test and to determine symmetries of the movements, a conventional line was set—the bisector deviation (BD) of the angle between the two extreme ranges of motion, i.e., the TPHAleft and TPHAright (Fig. 6). BD, initially transposed to the left in 86% of the subjects, was shifted to the sagittal axis of the body following mobilization (mBD). Before the mPNF, the bisector deviation between the TPHAleft and TPHAright was transposed to the left from the sagittal axis of the body by 4.73° (± SEM 0.50), and after mPNF, the shift to the left was by 1.35° (± SEM 0.37). This change was statistically significant (*p* < 0.001). The biggest shift of BD was noted in girls with scoliosis deeper that 35°.

Taking into account Akaike information criterion (AIC), the model with the normal distribution of identity link function was the most appropriate multivariate GLM model. In this model, repeated measurements were represented by a factor affecting the analyzed parameters (ATR, TPHA, BD). This factor was significant in models both with and without other factors such as Th Cobb, L Cobb, a brace, physiotherapy, and Risser test. That means that the mPNF influenced significant changes in the ATR, TPHA, and BD.

## Discussion

In the present research, it was revealed that a single use of unilateral mPNF significantly decreased the ATR, increased the TPHA range of motion, and improved motion symmetry reflected in the shift of BD to the sagittal axis of the body in girls with double idiopathic scoliosis. A multivariate analysis confirmed a considerable influence of mPNF on the changes of the ATR, TPHA, and BD. Additionally, the influence of mPNF was also considered significant when other parameters such as curve size in the thoracic and lumbar segment, Risser test, a brace, and physiotherapy were taken into account.

A significant improvement in the ATR values in the thoracic and lumbar spine after mobilization was observed in the study group. However, caution in the interpretation of the results is advised since we do not know whether these differences are not only statistically but also clinically significant. In 37 subjects, a difference of 0°–2° was found in the thoracic or lumbar spine after mPNF and such a change may be seen as clinically insignificant. Nevertheless, a greater improvement was noted in the majority of the subjects. It is also worth noting that in a number of girls, the ATR values decreased both in the thoracic and in the lumbar segment, which may affect the way clinical significance of these changes is seen. The biggest changes were noted in girls with scoliosis deeper than 35°.

In the literature of the subject, the issue of an immediate effect of particular techniques on the ATR values in scoliosis has not been frequently discussed. No publication has been found which would describe the application of muscle mobilization similar to the one used in our research. Wnuk et al. revealed an immediate positive effect of derotation manual therapy on a decrease in the ATR in two groups of girls with single and double scoliosis. The greatest effect reflected by the decreased ATR was achieved in girls with single scoliosis [17]. These results cannot be directly compared to our research results due to a different type of mobilization technique applied in the studies.

Derotation techniques are frequently used by manual therapists, chiropractors, and osteopaths. Certain studies indicate the effectiveness of treating scoliosis with manual therapy techniques, including derotation techniques [18, 19], but the authors of other studies are not certain about the long-term effects of manual therapy or chiropractic [20, 21]. Morningstar et al. revealed an improvement in radiological image in the group of 22 subjects aged 15–65 with scoliosis after a 4- to 6-week-long manipulative and rehabilitation therapy [18]. Lewis et al. applied Active Therapeutic Movement Version 2 device and home exercises using the Mulligan’s mobilization for 4 weeks and revealed an improvement in body position in 43 individuals with scoliosis from various age groups [19]. On the other hand, following a literature review, Romano and Negrini revealed that the level of the previous studies does not make it possible to draw conclusions regarding the effectiveness of manual therapy in treating scoliosis [21].

A change in the TPHA ranges was another effect of the applied mPNF. The value of the TPHAright in the study group before mobilization was significantly lower than the value of the TPHAleft. After the application of mPNF, the ranges of motion of the TPHA changed but the difference between the TPHAleft and TPHAright remained significant although it was smaller.

The noted limitation of the TPHAright in relation to the TPHAleft in the study group is in line with earlier findings reported by Stępień et al. [7]. This earlier study, conducted with the use of computer-assisted motion analysis on a group of adolescent girls with double scoliosis, found significant differences between ranges of motion in the transverse plane. The trunk rotation to the right was greater than the trunk rotation to the left, and the measured rotation of the pelvis to the left was significantly greater than the rotation to the right. The ranges of trunk rotation to the left and pelvis rotation to the right were significantly smaller in scoliotic girls than in their healthy peers [7].

Asymmetry of spinal rotation in the girls with scoliosis was also demonstrated in the latest studies using the TPHA test. In the research including 49 adolescent girls with double scoliosis and 49 healthy girls, a significant limitation in the TPHAright was noted in the group of girls with spinal deformity. The range of motion of the TPHAright was significantly lower not only than the range of motion of the TPHAleft in this group but also than the range of motion of the TPHAright noted in the group of healthy girls. A significant limitation of the range of motion of the TPHAright compared to the TPHAleft was also noted within the group of girls without scoliosis [8], which indicates the existence of a certain physiological asymmetry of rotational movements that increases in individuals with double scoliosis. The bilateral lower limb patterns used in unilateral mPNF were selected due to the determined direction of the limitation of the TPHAright.

An impairment of rotation in adolescent girls with progressive idiopathic scoliosis was also observed in much earlier studies conducted by other authors. Poussa and Mellin, who studied spinal mobility in 71 girls with idiopathic thoracic scoliosis, noted lower ranges of rotation in girls with a greater curvature of the spine. The authors concluded that decreased thoracic rotation and the straightening of the spine may serve as factors affecting the progression of scoliosis in the thoracic segment of the spine [6]. In another research on the girls with idiopathic scoliosis, McIntire et al. found that the strength of rotation towards the concavity of their primary curve was lower than the strength of rotation towards the convex side. The authors concluded that factors which might potentially lead to differences in the strength of muscles participating in rotation included soft tissue tension and limited rotation range [22].

To date, few authors have analyzed an influence of the therapy on an increase in the spinal range of motion. In their research, Lewis et al. noted an improvement in spinal ranges of motion in all directions except for flexion and extension. However, the research included subjects from a broad range of age groups, which made it difficult to analyze and compare the results [19].

An improvement in ROM in patients with scoliosis should be carefully analyzed. Usually, increased mobility of the spine is thought to be dangerous in scoliosis [4, 5, 23]. Czaprowski et al. found that generalized joint hypermobility occurred significantly more often in girls and boys with scoliosis compared to their healthy peers [5]. In their study, they used the Beighton scale which assesses mobility of the spine in the sagittal plane and does not take into consideration motion in the transverse plane. Generalized joint laxity and a higher incidence of scoliosis were found in rhythmic gymnastics trainees [4] and ballet dancers [23], but rotation was not assessed in these groups, either. To sum up, it should be concluded that the process of increasing spine mobility in scoliotic patients should be conducted carefully and further research in this field is necessary. In our opinion, it should be established every time in which plane a limited or increased range of motion of the spine occurs.

It is difficult to say what mechanism was responsible for an immediate effect in our research. There is a lot of evidence leading to the conclusion that the decreased ATR and the increased TPHA result from muscle relaxation, but muscle tension was not assessed in our study. A number of earlier studies revealed differences in the activity of paravertebral muscles between the concave and the convex sides of the curve in the subjects with scoliosis [24, 25]. A correlation was found between muscle function asymmetry and axial rotation of the spine [24]. In the present study, the muscle contract–relax technique was used, which in addition to the “hold–relax” technique, is utilized by PNF to relax tense muscles [12, 13]. Several authors have demonstrated the effectiveness of PNF muscle relaxation techniques [11, 26–29], but in none of the studies, an attempt was made at improving spinal rotation. It was observed that maximal muscle contraction is not required to achieve muscle relaxation and increase the range of motion [30–32]. For this reason, in the present study, the  physiotherapists used moderate resistance. The technique of contracting muscles for 5 s used in our study did not differ from that proposed by other authors [11, 30, 33], while the number of repetitions of muscle contractions chosen by the present authors was based on their earlier observations and clinical experience.

In the present study, the study group included all the girls with double scoliosis who were in the required age group, regardless of the results of the TPHA test conducted prior to the test. It results from the fact that at this stage of research, it is difficult to define what values of the TPHAleft and TPHAright should be seen as a norm and what values indicate movement limitation. Determining the norm requires further research on bigger samples including healthy individuals and subjects with various types of scoliosis. In the examined group of 83 girls with double scoliosis, there were 12 girls who did not reveal lower values of the TPHAright. This subgroup mainly included girls with non-progressive low scoliosis and low to moderate scoliosis. Two girls had scoliosis of over 30°. After the application of unilateral mPNF in this subgroup, an improvement in the ATR and a change of the TPHA values were noted in all the girls. The results indicate that there exists a certain mechanism, apart from a unilateral movement limitation, which affects the body position in scoliosis. The mechanism which underlies the improvement of the ATR and the increased mobility is not fully understood and needs further investigation.

In the past, rotation limitations were seen as one of the main factors affecting the development of idiopathic scoliosis [9, 10]. Burwell et al. stated that scoliosis results from a cyclical failure of mechanisms of rotation control in the trunk during gait [9]. Wong concluded that thoracic rotational instability leads to the development of scoliosis [10]. Asymmetries of rotational movements noted in the present research may serve as a completion of the aforementioned theories.

To sum up, it can be concluded that an improvement in the ATR, TPHA, and BD parameters in this research indicates an immediate effect in girls with double scoliosis. However, it does not prove the effectiveness of the undertaken actions. Therefore, further long-term observation and radiological confirmation are needed. According to the international guidelines, such long-term effectiveness is perceived as a higher satisfaction with appearance and a better quality of life in scoliotic adolescents, arrested progression of deformity, alleviation of pain and improved respiratory function and exercise efficiency [34].

### Limitations of the study

The study has certain limitations. It was revealed that an immediate effect of mPNF occurred only in the girls with double idiopathic scoliosis. An immediate effect seen as a decrease in the ATR and an increase in the TPHA with the reduction in the differences between the right and left sides does not prove the effectiveness of the method used since that would require long-term observation confirmed by radiographic evidence. It is recommended to assess the long-term efficiency of physiotherapy. In order to confirm the efficiency of the applied interventions, further studies are needed with a control group in which a different stretching method would be applied. Another obvious limitation is the fact that the ATR and TPHA measurements were performed by the physiotherapists applying mPNF.

The results of the study should be considered as a preliminary observation which allows better understanding of the spinal biomechanics in double idiopathic scoliosis. Although the effect achieved may be clinically insignificant, it may indicate a promising direction in the physiotherapy of scoliosis.

## Conclusions

Unilateral mPNF has an impact on the reduction in the angle of trunk rotation, improvement in the range of motion, and the symmetry of mobility in the transverse plane in the trunk-pelvis-hip complex in adolescent girls with double idiopathic scoliosis. The effects should be treated only as immediate. Further studies are required to determine long-term effects of mPNF on the spinal alignment.

## Abbreviations

AIS: Adolescent idiopathic scoliosis
ATR: Angle of trunk rotation
ATRL: Angle of trunk rotation in the lumbar spine
ATRT: Angle of trunk rotation in the thoracic spine
BD: Bisector deviation
L: Lumbar
mBD: BD after mPNF
mPNF: PNF mobilization
mTPHA: TPHA after mPNF
PNF: Proprioceptive Neuromuscular Facilitation
PT: Physiotherapist
ROM: Range of motion
SD: Standard deviation
SEM: Standard error of measurement
T: Thoracic
TPHA: Trunk-Pelvis-Hip Angle
TPHAleft/TPHAright: TPHA on the left/right
